# The Effectiveness of a Lactobacilli-Based Probiotic Food Supplement on Bone Mineral Density and Bone Metabolism in Australian Early Postmenopausal Women: Protocol for a Double-Blind Randomized Placebo-Controlled Trial

**DOI:** 10.3390/nu16081150

**Published:** 2024-04-12

**Authors:** Stephanie M. Resciniti, Jessica R. Biesiekierski, Ali Ghasem-Zadeh, George Moschonis

**Affiliations:** 1 Department of Food, Nutrition and Dietetics, La Trobe University, Bundoora, VIC 3086, Australia; s.resciniti@latrobe.edu.au; 2 Department of Nutrition, Dietetics & Food, Monash University, Notting Hill, VIC 3168, Australia; jessica.biesiekierski@monash.edu; 3 Department of Medicine and Endocrinology, Austin Health, The University of Melbourne, Heidelberg West, VIC 3081, Australia; alig@unimelb.edu.au

**Keywords:** study protocol, gut microbiota, postmenopausal women, probiotics, lactobacillus, bone mineral density

## Abstract

Osteoporosis affects one in three women over the age of 50 and results in fragility fractures. Oestrogen deficiency during and after menopause exacerbates bone loss, accounting for higher prevalence of fragility fractures in women. The gut microbiota (GM) has been proposed as a key regulator of bone health, as it performs vital functions such as immune regulation and biosynthesis of vitamins. Therefore, GM modulation via probiotic supplementation has been proposed as a target for potential therapeutic intervention to reduce bone loss. While promising results have been observed in mouse model studies, translation into human trials is limited. Here, we present the study protocol for a double-blind randomized controlled trial that aims to examine the effectiveness of three lactobacilli strains on volumetric bone mineral density (vBMD), trabecular, and cortical microstructure, as measured using High Resolution peripheral Quantitative Computed Tomography (HR-pQCT). The trial will randomize 124 healthy early postmenopausal women (up to 8 years from menopause) to receive either probiotic or placebo administered once daily for 12 months. Secondary outcomes will investigate the probiotics’ effects on areal BMD and specific mechanistic biomarkers, including bone metabolism and inflammatory markers. The trial is registered with Australian New Zealand Clinical Trials Registry (ACTRN12621000810819).

## 1. Introduction

Osteoporosis affects one in three women and one in five men over the age of 50 worldwide [1]. Increasing life expectancy in developed countries has brought osteoporosis and its clinical consequence, fragility fractures, into focus as a significant public health challenge [2]. Women in particular experience a pronounced rate of bone loss estimated to be 2–2.5% annually during the first five years following menopause [3,4], which then decreases to 1–2% annually a decade after menopause [5,6]. This rapid bone loss, primarily due to oestrogen deficiency post-menopause, is a key factor in the lower bone mass and heightened fracture risk observed in women compared to men [3]. The deficiency promotes osteoclastogenesis, leading to a negative imbalance in bone remodelling. Consequently, there is a progressive decline in bone mineral density (BMD) at critical osteoporotic fracture sites, including the lumbar vertebrae in the spine, femoral neck, and forearm [7].

Key lifestyle factors, particularly inadequate dietary intake of essential micronutrients, specifically calcium and vitamin D, are major contributors to bone loss [8]. Additionally, Vitamin K_2_ plays a significant role in bone metabolism [9,10,11,12], since it is essential for the γ-carboxylation of osteocalcin, a process crucial for bone formation and bone mineralization. Vitamin K is also synthesized by gut bacteria [13], underscoring the importance of gut microbiota in bone health. The human gastrointestinal tract’s microbiota performs vital functions, including immune regulation, maintenance of the gut barrier integrity, intestinal endocrine signalling, protection against pathogen overgrowth, and biosynthesis of vitamins, such as vitamin K_2_ [14,15]. These functions are integral to overall physiological health, including bone metabolism.

Pharmacological treatments for osteoporosis, while effective [16], suffer from low adherence, possibly due to fear of rare side effects [17] or complex dosage requirements [18]. This underscores the importance of exploring non-pharmacological options in preventing bone loss and reducing the risk of fragility fractures.

Recent evidence suggests that changes in the composition of gut microbiota through probiotic supplementation may favourably alter bone mass [19,20,21,22]. For example, in animal studies, a selected mixture of three probiotic strains (*Lactobacillus paracasei* DSM 13434, *Lactobacillus plantarum* DSM 15312, and *Lactobacillus plantarum* DSM 15313) [23] protected ovariectomized mice from ovariectomy-induced bone loss [20]. However, human clinical trials investigating the effect of probiotic supplementation on BMD are inconsistent. In a Swedish study [19], postmenopausal women receiving a similar probiotic *Lactobacillus* mixture experienced reduced areal BMD (aBMD) at lumbar spine, but no benefits at other sites, measured with dual energy X-ray absorptiometry (DXA), compared to placebo. Conversely, a different probiotic strain supplement administered to older (75–80 years old) postmenopausal women, showed increased volumetric BMD (vBMD) (measured with high-resolution peripheral quantitative computed tomography (HR-pQCT)) at the distal tibia, compared to the placebo [21].

To the best of our knowledge, there are only five clinical trials that have investigated the effectiveness of probiotic supplementation on BMD in postmenopausal women [19,21,24,25,26] to date. Their findings were assessed in two systematic literature reviews [27,28] that reported modest improvements in BMD at the lumbar spine, but no changes at other skeletal sites (particularly hip BMD). Only one trial examined vBMD using the highly sensitive HR-pQCT measure [25], whereas the remaining five studies measured aBMD via dual-energy X-ray absorptiometry (DEXA), a less sensitive but more widely available bone mineral density assessment method. Furthermore, inconsistencies in the existing evidence could be attributed to variations in probiotic strains, intervention durations (6–12 months), diversity in reported bone density measures, participants’ age (mean age ranging from 51–76 years), years from menopause (up to 12 years or more), underpowered or low sample sizes, and discrepancies both in reported bone turnover markers (BTM) and other primary biomarkers (what was measured in the findings reported).

To address these variations in the evidence, the current study examines a probiotic using a combination of strains with demonstrated efficacy in both a mouse model [23] and a human trial in postmenopausal women [19], utilises a 12-month intervention period, and employs aBMD, vBMD, and bone microstructure measurement techniques synergically. We specifically focus on early postmenopausal women (i.e., up to 8 years from menopause) to target the period of most rapid bone loss [3], examining a range of hormonal, inflammatory, and bone metabolism biomarkers, alongside changes in gut microbiota composition. The current study contributes to this emerging field by examining specific mechanistic biomarkers in early postmenopausal women.

Therefore, the primary aim of the study is to assess the effect of a probiotic containing three lactobacilli strains *Lactiplantibacillus plantarum* HEAL9 (HEAL9™), *Lactiplantibacillus plantarum* HEAL19, and *Lacticaseibacillus paracasei* 8700:2 (previously identified as *Lactobacillus paracasei* DSM 13434, *Lactobacillus plantarum* DSM 15312, and *Lactobacillus plantarum* DSM 15313) once daily for 12 months on vBMD in healthy early postmenopausal women, compared to a control group that will receive a placebo. Secondary aims are to assess changes between groups over 12 months in aBMD, gut microbiota composition, blood biomarkers (including bone metabolism and turnover markers, inflammatory markers), and dietary intake. The expected intervention effect is proposed to be mediated by beneficial changes in gut microbiota composition that stimulate bone formation, suppress osteoclast differentiation, and reduce inflammation, as well as improve markers of bone mineralisation, including calciotropic hormones and increased gut production of vitamin K_2_.

The study hypothesizes that supplementation with this specific probiotic once daily for 12 months will have more favourable effects on vBMD, aBMD, and bone metabolism, compared to the placebo. This effect is expected to be mediated by beneficial changes in gut microbiota composition (i.e., shift in bacterial diversity) and a consequent production of molecules that impact bone mineralization, calcium metabolism, osteoclastogenesis, and immune function inflammation (i.e., lower inflammatory biomarkers).

## 2. Materials and Methods

### 2.1. Study Design

The design is a double-blind randomized controlled trial that examines the effectiveness of a probiotic supplement, containing three Lactobacillus strains, versus placebo in a 1:1 ratio in healthy early postmenopausal women. The product will be taken daily for 12 months. This study was approved by the La Trobe University Human Research Ethics Committee (HREC 21038) and will be conducted at the Food, Nutrition and Dietetics Research Laboratory located at La Trobe University, Bundoora, Australia. The protocol was designed according to Standard Protocol Items: Recommendations for Intervention Trials (SPIRIT). Trial registration: Australia & New Zealand Clinical Trials Registry (ACTRN12621000810819).

### 2.2. Participants

**Figure 1:**
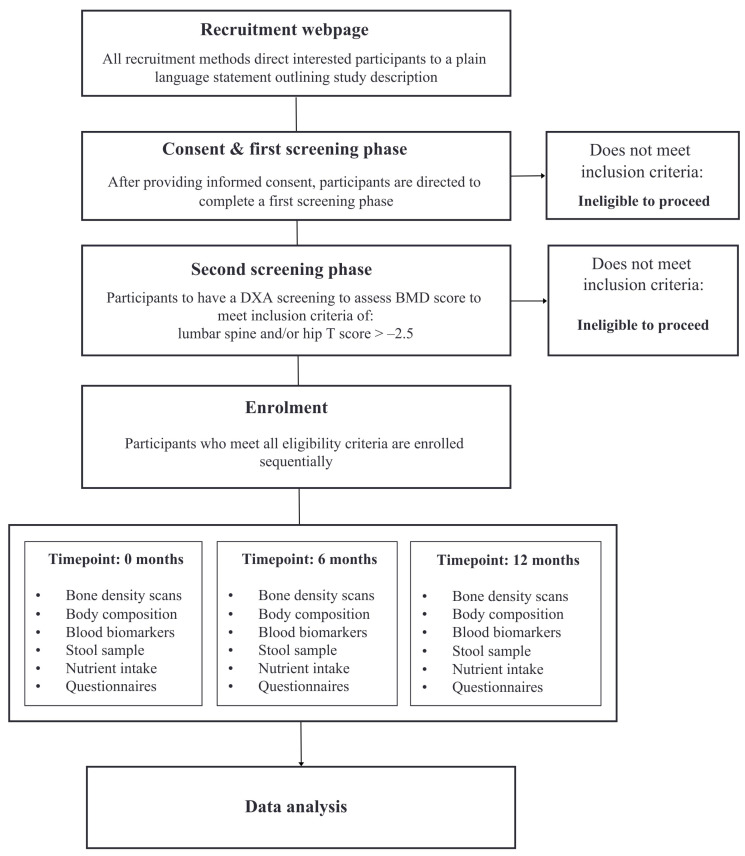
Flowchart of recruitment and enrolment process.

Healthy early postmenopausal women between the ages of 45–65 years will be recruited to participate. Participants must have a minimum of at least one year since their last menstruation to meet the definition of menopause, and a maximum of up to eight years since menopause to meet the definition of early menopause. Participants will be eligible to participate in the study if they are a non-smoker, not taking hormone replacement therapy (or menopausal hormone treatment) for at least 12 months, have a body mass index (BMI) of between ≥18 and ≤32 kg/m^2^, and have maintained stable body weight for at least six months. The exclusion criteria include: a history of bone disease or any medical conditions that influence bone health or inflammation, such as hypothyroidism, inflammatory bowel disease, and rheumatoid arthritis; malignancy within the last five years; any systematic medication such as glucocorticoids; or consumption of more than four standard units of alcohol per day or more than five cups of coffee per day. Women will be further assessed at screening and eligible for enrolment with a T-score of >−2.5 at the total hip or lumbar spine (L1–L4) as measured using DEXA. If a screened participant’s T-score is ≤−2.5, this participant will be referred to their health practitioner for further investigation. Participants must be willing to give informed consent. Study visits will take place at La Trobe University, Bundoora, VIC, Australia, as well as the Austin Repatriation Hospital, Heidelberg, VIC, Australia. The study will be advertised by several methods, including distribution amongst professional networks (to staff and students at La Trobe University), email lists, and via targeted social media advertising circulated via platforms including Facebook, Instagram, and Twitter. Refer to Figure 1 for the recruitment process.

### 2.3. Randomization and Blinding

A computer-generated 10-block randomization list assigned study IDs sequentially to group allocations. An independent researcher, not involved in the trial, used this list to determine the probiotic group assignment. This randomization list was then provided to the manufacturer of the product (not involved in data collection) to box and label the product (either probiotic or placebo) in sequentially numbered containers. Each container was identical regardless of group allocation. These identical boxes were then provided to the research team. Neither the researchers nor the participants were aware of the group allocation. Participants will be assigned a study ID in the order of enrolment. The probiotic and placebo will have identical appearance and flavour.

### 2.4. Intervention

The active investigational product (IP) is a combination of the three probiotic bacteria *Lactiplantibacillus plantarum* HEAL9 (HEAL9™), *Lactiplantibacillus plantarum* HEAL19, and *Lacticaseibacillus paracasei* 8700:2, delivered in a capsule containing a powder with freeze-dried bacteria and maltodextrin used as filler. Each bacterial strain is equally represented, and the total bacterial dose is 1 × 10^10^ CFU/capsule to be taken daily for 12 months with water (food optional). The placebo capsules will have the same visual appearance with the same taste and texture as the active IP, with the exception that the probiotic powder is substituted with some yeast peptone. Adherence to intake will be evaluated by counting the number of unused capsules returned.

### 2.5. Restrictions during the Study

Participants will be asked to refrain from using other products containing probiotic bacteria, maintain stable body weight during the study (defined as weight changes that do not exceed ±5 kg), be fasting overnight (10 h) before all clinic visits including baseline (water allowed, but alcohol not allowed within 24 h prior to visit), refrain from strenuous exercise (defined as greater than 70% of the maximal pulse rate for one hour or more, or 24 h prior to and during the day of each clinic visit). Caffeine consumption must be limited to five cups of coffee, or corresponding amount of other caffeine-containing products, per day throughout the study period. Participants must refrain from smoking or use of nicotine-containing products, blood or plasma donations, or using calcium or vitamin D supplements or hormonal therapy for the study duration. Participants will be instructed to maintain their current physical activity habits throughout the duration of the study, unless their healthcare providers advise them differently. Once enrolled, participants who no longer meet the inclusion criteria or who can no longer comply with the restrictions will be terminated from the study. Unblinding will not take place until after the analysis is complete, even for participants who have terminated early.

### 2.6. Outcomes

#### 2.6.1. Bone Mineral Density Measurements

The primary outcome is the change in cortical and trabecular vBMD as measured by high resolution peripheral quantitative computed tomography (HR-pQCT, Xtreme CT; Scanco Medical, Brüttisellen Switzerland) at the distal tibia and distal radius after 12 months of intervention compared to placebo. Participants will be scanned at baseline (Time point: 0) and 12 months (Time point: 12 month) to assess changes.

Scans will be performed by a trained researcher in HR-pQCT scanning and interpretation, according to the established guidelines and manufacturer’s instructions [29]. Briefly, this involves the upper and lower limbs being positioned in anatomically formed carbon fibre casts provided by the manufacturer (to minimize limb motion during scanning). Once the limb is placed into the gantry of the scanner, a 2D scout view is obtained to select the region of interest for the 3D measurement. Using the fixed offset method, which is suitable for follow-up studies, the operator will place a reference line at the inflection point on the endplate of the distal radius or tibial plafond. The scan region begins 9.5 mm and 22.5 mm proximal to the reference line for the radius and tibia, respectively. The HR-pQCT images will be analysed with specific software developed by the manufactures. A semi-automated slice-by-slice contouring process will be utilized to identify the periosteal boundary of the bone, thereby extracting the bone region from the surrounding soft tissue. The delineation of the cortical and trabecular compartments will then be performed automatically using a filter and threshold-based algorithm.

Additional bone mineral density measures include changes in aBMD measured by a trained DEXA researcher at the following sites: spine (L1–L4), total hip, femoral neck, and forearm, using a Hologic DXA machine (Time points: 0, 6, and 12 months). Following the manufacturer’s and standard guidelines, the non-dominant hip and forearm will be assessed using DXA. In the presence of disorders such as fragility fractures in the non-dominant site, the opposite site will be scanned. All scans will be analysed by one person to minimize intra-operator errors. A study that will assess the coefficient of variation in DXA measurements will be performed using 30 participants scanned twice. Calcaneal (heel) measurements will be carried out using quantitative ultrasound (QUS, Achilles, Abingdon, UK) and measured at time points: 0, 6, and 12 months.

#### 2.6.2. Gut Microbiome Composition

Gut microbiome analysis will be performed on participant-collected stool samples. Participants will collect samples at home according to manufacturer’s instructions (Invitek, Berlin, Germany) prior to each study visit and return it to the lab for storage at −20 degrees centigrade. All samples will be analysed using 16S sequencing techniques using QIAGEN DNeasy Power Kit (Qiagen 12888, Hilden, Germany). Changes in profile, such as microbiome alpha diversity (richness, Shannon Diversity Index), phylogenic beta diversity, relative abundance of taxa, and functional potential from baseline to 12 months, will be investigated, and will also function as a compliance check for the intervention group to verify intake.

#### 2.6.3. Blood Biomarker Measurements

Blood samples will be drawn using standard sterile IV venipuncture techniques by a trained phlebotomist. All samples will be obtained from a vein in the antecubital fossa of the arm using standard phlebotomy procedures. Plasma and serum samples will be decanted and aliquoted into de-identifiable labelled tubes and stored in a −80 °C freezer for subsequent analysis. Serum and plasma will be processed, stored, and assayed in the La Trobe Nutrition Clinical Trial Laboratory. Deidentified samples will also be sent to accredited private pathology laboratories for further analyses for the measurement of the concentrations of blood biomarkers (Time points: 0, 6, and 12 months). The analyses of serum or plasma for all other biomarkers will be conducted after data collection is complete, using standardised procedures and assays. This includes bone formation (Procollagen type 1 N propeptide (P1NP), total and undercarboxylated osteocalcin) and blood–bone resorption (Serum type I collagen cross-linked C-Telopeptide (CTx) or Tartrate-resistant acid phosphatase 5b (TRAP 5b)); osteoclast differentiation molecules osteoprotegerin (OPG) and receptor activator of nuclear factor kappa-Β ligand (RANKL); calciotropic hormones 25-hydroxy vitamin D, 1,25-hydroxy vitamin D, parathyroid hormone (PTH), and calcitonin; renal and liver function markers to assess normal function; and inflammatory markers high sensitivity C-reaction protein (hsCRP) and interleukin-6 (IL-6).

#### 2.6.4. Dietary Intake

Dietary intake will be measured using 3-day food diaries at each time point, and analysed using FoodWorks 10 (Xyris software), the leading nutrition analysis software in Australia. Macro and micronutrient intake values will be derived from 3-day food diary analysis. Participants will be asked to record all food and drink intake for two weekdays and one weekend day that are typical of their usual diet. A validated food frequency questionnaire (FFQ), titled Comprehensive Nutrition Assessment Questionnaire [30], will also assess dietary intake and patterns, as well as fermentable carbohydrates at each timepoint. This FFQ was selected given its identification and quantification of fermentable carbohydrates (i.e., FODMAPs), known to impact gut microbiome composition [31].

#### 2.6.5. Body Composition and Anthropometry

Body composition will be measured using DXA (Hologic) by a trained researcher. Participants will attend all study visits fasted and at approximately the same time of day per timepoint to avoid variation. Body composition will also be measured using bioimpedance analysis (BIA) with the Tanita MC-780 analyser (Poznań, Poland). Body weight and standing height is measured by using a digital scale (Seca, Hamburg, Germany) with an accuracy of ±100 g and a commercial stadiometer (Leicester Height Measure; CMS Instruments, Oxford, UK) to the nearest 0.5 cm, respectively, while participants wear light clothing and no shoes.

#### 2.6.6. Self-Reported Questionnaires

Data collected through questionnaires will be self-reported, unless indicated where obtained with an interview with study participants. The questionnaires, administered via REDCap, will collect information on:

Demographics: The socio-demographic questionnaire will collect information on participants’ socio-demographic characteristics, i.e., date of birth, years of education, employment status, occupation, country of birth, marital status, ethnicity, and areas of residence (Time points: 0).

Medical History: Medical and surgical history will be obtained with an interview in order to verify that the eligibility criteria are met. Any medication used during the study (concomitant medications) will be recorded in the study diary/CRF. Concomitant medications will be coded according to the WHO Anatomic Therapeutic Chemical (ATC) classification system (Time points: 0).

Fracture Risk: The Fracture Risk Assessment Tool (FRAX^®^) (online version adopted to the Australian population) [32] will be used to calculate the 10-year probability of fracture without BMD. This calculation is based on age, weight, height, alcohol consumption, history of osteoporosis, and several other risk factors. FRAX^®^ was developed by the WHO, and is managed by the University of Sheffield in the UK. (https://www.sheffield.ac.uk/FRAX/tool.aspx, accessed on 1 July 2021) (Time points: 0).

Physical Activity: The Active Australia Questionnaire (AAQ) has an acceptable validity and reliability for recording physical activity levels in Australian middle-aged women [33]. Study participants will report the frequency and duration of time in the previous week spent walking briskly, in moderate-intensity leisure time physical activities, and in vigorous-intensity physical activities (Time points: 0, 6, and 12 months).

Sunlight Exposure: Data on sunlight exposure will be collected via the use of a validated questionnaire [34] that will record information on study participants’ personal ultraviolet B (UV-B) radiation exposure (i.e., time of day, latitude, and season) and modifying factors (i.e., cloud, tree cover, surface, clothing, and sunscreen use). Data on the dose of UV-B radiation in the areas of residence of study participants will also be extracted from the online records of the Australian Radiation Protection and Nuclear Safety Agency (Time points: 0, 6, and 12 months).

Participants will be instructed to report adverse events at the time of event. If it is determined that the intervention is not causative of the adverse event, the participant will be allowed to maintain their participation in the study. Further assessment of any adverse events will be examined as to whether these differ between intervention and control groups.

Table 1 below shows an overview of study data collection and time points.

### 2.7. Sample Size

The sample size calculation was based on the changes observed in total tibia volumetric BMD (vBMD) between the two treatment arms (statistical power 80%, probability of type I error 0.05) and is adequate to achieve a difference of 1% (standard deviation 1.78%) in a previously published study [21]. This estimation showed the need to recruit 50 study participants per treatment arm. Expecting a drop-out rate of 20% from baseline to follow-up measurements, an additional sample of 24 study participants will be recruited, thus leading to total sample size of 124 (i.e., 62 per treatment arm).

### 2.8. Statistical Analysis

All continuous variables will be checked for the normality of their distribution. Normally distributed variables will be presented as Mean (standard deviation), while non-normally distributed ones will be presented as Median (interquartile range). General linear models (Repeated Measures Analysis of Variance) will be used to examine within-group changes in the majority of primary and secondary outcome measures across all time points (baseline, 6-, and 12-month follow-up) separately for each group (probiotic and placebo), as well as between-group differences at each time point. Within-group changes across all time points (baseline, versus post-intervention, versus follow-up) and between-group differences in each time-point will be also examined for biological sample data using the same statistical test. Appropriate corrections will be applied for post hoc multiple comparisons, while all analyses will be adjusted for potential confounding factors (e.g., age, body mass index, sunlight exposure, etc.). Correlations will also be used to explore the relationship between questionnaires and biological sample data.

Mediation analysis will also be conducted to examine the potential mediating role of changes in gut microbiota composition, and to examine the potential mediating role of other biological markers on the changes observed in BMD and bone metabolism indices. This will be based on a multiple regression model between the intervention, the outcome, and the potential mediators (e.g., a one of the mediation tests will assess the effect of the intervention on vBMD, examining the changes in serum concentrations of inflammatory markers as potential mediators of the intervention’s effect). The analysis will provide a % mediating effect, i.e., the % of the variability of the examined outcome explained by the examined mediating factors.

All statistical analyses will be performed using the SPSS statistical analysis software version 29 for Windows. Both Per-Protocol (PP), and Intention-to-Treat (ITT) analyses will take place. Per-protocol analysis will compare treatment groups, including only those study participants who completed the treatment that was originally allocated to them. Adherence to the intervention will also be taken into consideration in the per-protocol analysis by identifying and excluding potential non-adherent study participants from the analysis. All reported *p*-values will be two-tailed, while the level of statistical significance will be set at *p* < 0.05.

## 3. Results

The study remains ongoing, and as such, results are not yet available to be reported as part of this study protocol paper.

## 4. Discussion

This study aims to assess the effectiveness of gut microbiota modulation on bone mineral density and bone microstructure in early postmenopausal women compared to placebo. Preliminary mouse model results demonstrated that probiotics improve bone density [35,36], but translation in human clinical trials has produced inconclusive results. Of the five randomized clinical trials that measured aBMD at lumbar spine, total hip, and/or femoral neck, only one reported improvement at more than one site [25]. Jansson and colleagues (2019) reported improvements in spine but not femoral neck, and no differences in total hip [19], whereas Takimoto and colleagues (2018) reported improvements at hip, but no differences in spine [26]. Nilsson and colleagues (2018) reported no differences in aBMD at either lumbar spine or total hip, but did report differences in vBMD (describing bone geometry and structure) favouring the probiotic intervention group [21]. Indeed, only one of the published trials [19] has been adequately powered to demonstrate aBMD differences, which may account for the inconsistency in results. Furthermore, two [24,26] trials had sample sizes (*n* = 76 and *n* = 50, respectively) powered to detect changes in specific blood biomarkers rather than BMD. One trial [26] provided a power calculation, but did not specify which outcome measure was used to determine effect size. This indicates that adequately powered studies combined with appropriately sensitive measurement techniques are required to better elucidate the effect of probiotics on bone density in humans.

In addition to adequately powered trials, sufficient intervention duration is required to detect changes in bone mineral density [37]. Two [24,26] of the five reported trials had an intervention duration of 6 months, which may be insufficient to detect change in aBMD. Intention to treat analysis was used in both analyses due to dropouts, further indicating that sample size and short duration may be responsible for no differences detected between groups. The current study is a 12-month intervention, and has a sample size of *n* = 124.

Jansson and colleagues (2018) were the only authors to identify years from menopause as part of their inclusion criteria (up to 12 years from menopause) [19]. Other authors only identified postmenopausal women and specified ages ranges for inclusion (for example, 50–72 years or 60–85 years). The period immediately following menopause has been identified as the time of greatest bone loss [4], which accounts for the higher prevalence of osteoporosis in women. This presents a novel opportunity to specifically target this timeframe for intervention. Indeed, Jansson and colleagues demonstrated that when time from menopause was limited to 12 years in the inclusion criteria, the intervention group showed less aBMD loss at lumbar spine compared to placebo. When a subgroup analysis limited year from menopause to 6 years, the probiotic group experienced less than 0.2% mean aBMD loss, whereas the placebo group reported 1.2% mean aBMD loss (*p* = 0.016). Interestingly, participants that were more than 6 years from menopause in the probiotic group experienced an improvement of 0.2% mean aBMD. This might indicate that benefits of probiotic intervention may have a role to play in the retention or improvement of bone density regardless of years from menopause. Nevertheless, the current study has limited years from menopause to 8 years, but also included a general age range (45–65 years) to further elucidate this phenomenon.

The strengths of the current study protocol are: the inclusion criteria targeting women with no more than eight years from menopause; strict exclusion criteria, such as limiting years from menopause; robust study design using novel and highly sensitive measurement techniques such as BMD, through HR-pQCT in addition to DXA, well-established microbial analysis techniques, and the inclusion of key biomarkers for mechanistic examination of bone density changes; and an a priori analysis plan. A limitation of the study is that given we are recruiting from a healthy population, the cohort will likely have a healthy user bias and may not be applicable to other populations such as those with a higher BMI, women more than eight years from menopause, or men. Furthermore, some self-reported measures may be subject to recall bias, such as dietary intake, physical activity, and years from menopause, as these are unable to be objectively evaluated. However, these limitations are present in all self-reported measures and are not unique to this trial. Limitations of the nutrient data have been mitigated by including two rather than one reported measure. All questionnaires will be completed during study visits, and participants have the capacity to clarify questions to avoid misunderstanding, limiting the potential for incorrect data being reported for physical activity and other measures. Due to the ad hoc nature of menstruation during menopause, the exact date of cessation may not always be able to be determined. Therefore, the appropriate year of cessation will be determined to be sufficient to classify eligible women as ‘postmenopausal’.

Reducing the burden of fragility fractures later in life would improve quality of life. The International Osteoporosis Foundation highlights the need to prioritize prevention as a key strategy for managing fragility fractures [38]. The findings of this study have the potential to facilitate the retention and potential improvement of BMD during a critical period of bone loss for postmenopausal women whilst elucidating mechanistic pathways of action for gut microbiota modulation.

## Figures and Tables

**Table 1:** Overview of study data collection and timepoints.

	Screening Phase	Timepoint 0	Timepoint +6 Months	Timepoint +12 Months
Screening				
Informed consent	x	x		
Inclusion/exclusion criteria	x			
DEXA (spine or total hip)	x			
Data Collection				
Informed consent	x			
Bone density measurements				
DEXA (spine, total hip, femoral neck, forearm), QUS		x	x	x
HR-pQCT (tibia, radius)		x		x
Stool sample collection and storage (−20 °C)		x	x	x
Blood collection, processing, and storage (−80 °C)		x	x	x
Body composition				
DEXA (whole body), BIA		x	x	x
Nutritional Intake				
3-day food diary, FFQ		x	x	x
Questionnaires				
Physical activity (AAQ), Sun exposure, Quality of Life, Supplement use		x	x	x
Socio-demographics, FRAX		x		

DEXA—Dual energy X-ray absorptiometry, QUS—Quantitative Ultrasound, HR-pQCT—High Resolution peripheral Quantitative Computed Tomography, BIA—Bioimpedance Analysis, FFQ—Food Frequency Questionnaire, AAQ—Active Australia Questionnaire, FRAX—Fracture Risk Assessment Tool.

## Data Availability

The data presented in this study are available on request from the corresponding author. The data are not publicly available due to privacy restrictions and conditions of ethical approval.
